# The Efficacy of Melatonergic Receptor Agonists Used in Clinical Practice in Insomnia Treatment: Melatonin, Tasimelteon, Ramelteon, Agomelatine, and Selected Herbs

**DOI:** 10.3390/molecules30183814

**Published:** 2025-09-19

**Authors:** Kacper Żełabowski, Wojciech Pichowicz, Izabela Skowron, Jagoda Szwach, Kamil Biedka, Michał Wesołowski, Katarzyna Błaszczyk, Oliwia Ziobro, Wiktor Petrov, Wirginia Kukula-Koch, Agnieszka Chłopaś-Konowałek

**Affiliations:** 1 Scientific Society for Psychopharmacology, Department of Forensic Medicine, Wroclaw Medical University, 4 J. Mikulicza-Radeckiego Street, 50-345 Wroclaw, Poland; kacper.zelabowski@outlook.com (K.Ż.); wojciech.pichowicz@outlook.com (W.P.); izabela.skowron@student.umw.edu.pl (I.S.); jagoda.szwach@student.umw.edu.pl (J.S.); oziobro@gmail.com (O.Z.); wiktor.petrov@student.umw.edu.pl (W.P.); 2 Department of Physiology and Pathophysiology, Division of Pathophysiology, Wroclaw Medical University, Chalubinskiego 10, 50-368 Wroclaw, Poland; kamil.biedka@umw.edu.pl (K.B.); michal.wesolowski@umw.edu.pl (M.W.); 3 Department of Physiology and Pathophysiology, Division of Physiology, Wroclaw Medical University, Chalubinskiego 10, 50-368 Wroclaw, Poland; blaszczykrzyk@gmail.com; 4 Department of Pharmacognosy with Garden of Medicinal Plants, Medical University of Lublin, Chodźki 1, 20-093 Lublin, Poland; 5 Department of Forensic Medicine, Division of Molecular Techniques, Wroclaw Medical University, Sklodowskiej-Curie 52, 50-369 Wroclaw, Poland

**Keywords:** insomnia, melatonin, tasimelteon, ramelteon, agomelatine, melatonergic receptor agonists, circadian rhythm, sleep–wake disorders, medicinal plants, herbs

## Abstract

Insomnia is a common and complex disorder, rooted in the dysregulation of circadian rhythms, impaired neurotransmitter function, and disturbances in sleep–wake homeostasis. While conventional hypnotics such as benzodiazepines and Z-drugs are effective in the short term, their use is limited by a high potential for dependence, cognitive side effects, and withdrawal symptoms. In contrast, melatonergic receptor agonists—melatonin, ramelteon, tasimelteon, and agomelatine—represent a pharmacologically targeted alternative that modulates MT1 and MT2 receptors, which are pivotal to the regulation of circadian timing and sleep initiation. Clinical evidence supports the efficacy of these agents in reducing sleep onset latency, extending total sleep duration, and re-aligning disrupted circadian rhythms, particularly among older individuals and patients with non-24 h sleep–wake disorders. Notably, agomelatine offers additional antidepressant properties through selective antagonism of the 5-HT_2C_ receptor in micromolar concentrations. In contrast, its agonistic activity at melatonergic receptors is observed in the low sub-nanomolar range, which illustrates the complexity of this drug’s interactions with the human body. All compounds reviewed demonstrate a generally favorable safety and tolerability profile. Accumulating evidence highlights that selected medicinal plants, such as chamomilla, lemon balm, black cumin, valeriana, passionflower and lavender, may exert relevant hypnotic or anxiolytic effects, thus complementing melatonergic strategies in the management of insomnia. This structured narrative review presents a comprehensive analysis of the molecular pharmacology, receptor affinity, signaling pathways, and clinical outcomes associated with melatonergic agents. It also examines their functional interplay with serotonergic, GABAergic, dopaminergic, and orexinergic systems involved in arousal and sleep regulation. Through comparative synthesis of pharmacokinetics and neurochemical mechanisms, this work aims to inform the development of evidence-based strategies for the treatment of insomnia and circadian rhythm sleep–wake disorders.

## 1. Introduction

The etymology of the term “insomnia” derives from Latin, combining “in” meaning “not” and “somnus,” meaning “sleep” [1]. While commonly interpreted as the simple absence of sleep, insomnia is a multifaceted condition. The International Classification of Sleep Disorders, Third Edition (ICSD-3-TR) [2], developed by the American Academy of Sleep Medicine, is the leading framework for the classification of sleep disorders. This updated version of ICSD-3 organizes sleep disorders into six major categories: Insomnia Disorders, Sleep-Related Breathing Disorders, Central Disorders of Hypersomnolence, Circadian Rhythm Sleep–Wake Disorders, Parasomnias and Sleep-Related Movement Disorders [2]. The definition of insomnia disorders presented by the American Academy of Sleep Medicine is consistent with the descriptions provided in the 11th Revision of the International Classification of Diseases (ICD-11) and the 5th edition of the Diagnostic and Statistical Manual of Mental Disorders (DSM-5) [3,4]. In these documents, insomnia disorders are characterized by difficulty falling asleep (onset insomnia), difficulty maintaining sleep (middle insomnia), or early morning awakenings (late insomnia) [3,5,6]. These disturbances occur despite adequate opportunity and conditions for sleep [6], and they result in general dissatisfaction with sleep quality and various daytime impairments. Common daytime symptoms include fatigue, low mood or irritability, general malaise, and cognitive difficulties such as impaired concentration or memory. Individuals reporting nighttime symptoms without associated daytime dysfunction are not classified as having clinical insomnia [4,6].

Insomnia is a highly prevalent medical condition that significantly impacts quality of life. Studies have demonstrated a direct correlation between inadequate sleep and an increased risk of developing metabolic diseases, such as diabetes, obesity, hypertension, cardiovascular disease, and depression [7,8,9]. These conditions can lead to consequences such as road accidents and death [10]. Severe sleep-related conditions affect approximately 10–15% [11] of adults and 25% of the elderly population. These conditions are associated with a substantial socioeconomic burden [12]. Up to 69% of patients at primary care clinics have insomnia, which is particularly prevalent among individuals with chronic medical conditions [13].

Although sleep difficulties are commonly encountered in everyday clinical practice, the problem is frequently underestimated, and patients often do not receive adequate care [14,15]. Treatment strategies for insomnia are determined by its etiology, duration, and severity of symptoms. Currently, several therapeutic approaches are recognized, encompassing both non-pharmacological and pharmacological strategies.

Non-pharmacological interventions, such as improving sleep hygiene by maintaining a regular sleep schedule, creating a conducive sleep environment, and avoiding stimulating activities before bedtime, should be the primary approach to managing insomnia. Behavioral interventions, such as relaxation techniques and cognitive behavioral therapy for insomnia, should also be considered first-line treatments. It can be used alone or in combination with herbal and natural supplements [16]. However, pharmacotherapy is often necessary in clinical practice.

Traditionally prescribed hypnotics, including benzodiazepines (e.g., diazepam, lorazepam, temazepam) and Z-drugs (e.g., zolpidem, zopiklon) [17] are associated with a significant risk of dependence [18], long-term deterioration in sleep quality [19] and the emergence of withdrawal symptoms upon discontinuation [20].

In the short term, antipsychotics may cause sedation, blurred vision, dizziness, a dry mouth, and urinary irregularities. In the long term, they may cause increased appetite and subsequent weight gain. Routine insomnia drugs cause daytime drowsiness and interfere with activities that require cognition and awareness. While these medications can be effective in the short term, they are associated with various side effects, including dependence, tolerance and adverse reactions. Furthermore, long-term use of these hypnotics may result in rebound insomnia and withdrawal symptoms when discontinued [21].

Consequently, there is growing interest in safer therapeutic alternatives that act through physiological mechanisms regulating the sleep–wake cycle.

Herbal and natural supplements have gained popularity as potential sleep aids due to their perceived safety, lower risk of dependence, and fewer side effects compared to conventional medications. These supplements often contain sedative, anxiolytic, or sleep-promoting compounds, such as flavonoids, terpenes, and amino acids [22,23].

**Figure 1:**
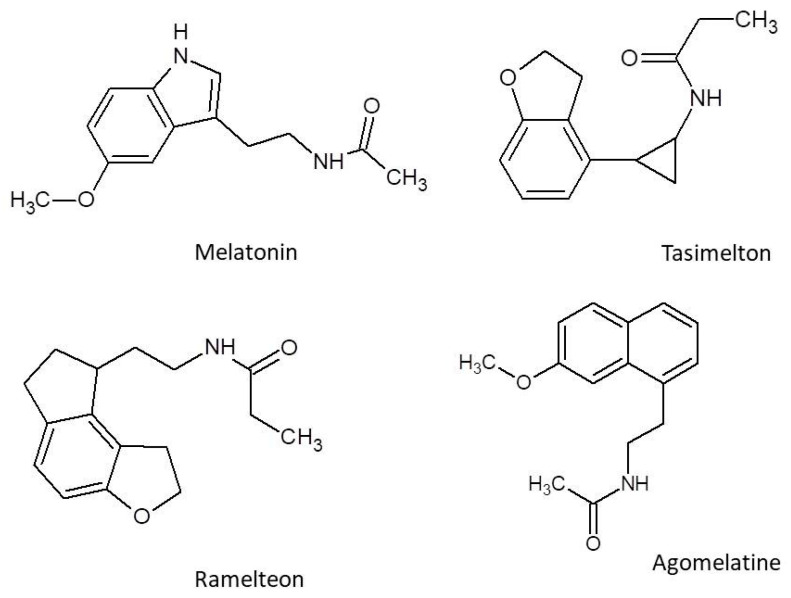
Structural formulas of melatonin, agomelatine, ramelteon, and tasimelteon.

Particular attention has been directed toward melatonergic receptor agonists—compounds that mimic the action of endogenous melatonin, a hormone involved in circadian rhythm regulation. This pharmacological group includes melatonin, agomelatine, ramelteon, and tasimelteon (Figure 1), which differ in their pharmacokinetic properties and receptor profiles but share a common therapeutic goal.

This review aims to provide a comprehensive overview of the current knowledge regarding clinical efficacy of selected melatonergic receptor agonists in the treatment of insomnia. Additionally, we examined the effectiveness of selected herbal supplements for managing sleep disorders by critically reviewing evidence from clinical studies on their efficacy and safety.

## 2. Methodology

This article constitutes a narrative review aimed at summarizing current clinical evidence on the efficacy and pharmacological characteristics of melatonergic receptor agonists and selected herbal agents in the treatment of insomnia and circadian rhythm sleep–wake disorders. To identify the most relevant clinical evidence on the efficacy of melatonergic receptor agonists in the treatment of insomnia and circadian rhythm sleep disorders, an extensive literature search was conducted across various academic databases: PubMed, Embase, Web of Science, and Scopus. The search strategy included both MeSH and Emtree terms and free-text keywords to ensure the most effective retrieval of relevant articles. MeSH terms used during PubMed screening: ‘melatonin’, ‘agomelatine’, ‘ramelteon’, ‘tasimelteon’, ‘receptors, melatonin’, ‘sleep disorders, circadian rhythm’, ‘insomnia’, ‘jet lag syndrome’, ‘sleep onset insomnia’, ‘maintenance insomnia’, ‘circadian misalignment’, ‘delayed sleep phase syndrome’, ‘non-24’, ‘shift work disorder’ and ‘jet lag disorder’. The Emtree terms searched in Embase included: ‘melatonin’, ‘melatonin receptor agonist’, ‘agomelatine’, ‘ramelteon’, ‘tasimelteon’, ‘insomnia’, ‘circadian rhythm sleep disorder’, and ‘jet lag’, ‘insomnia’, ‘sleep’, medical plant, ‘herbal medicine’, ‘valerian’, ‘passionflower’, ‘lavender’ or “Phyto-medical plant”. “insomnia” or “sleep” or “plant” or “herb” or “extract” in the title.

In addition, free-text keywords were included to capture relevant studies not yet indexed under standardized terms. These included: ‘melatonergic’, ‘MT1’, ‘MT2’, ‘non-24-h sleep–wake disorder’, ‘delayed sleep phase’, ‘sleep onset latency’ and ‘sleep efficiency’. Inclusion criteria: articles published after 2014 providing most recent data, clinical studies, performed on adults, studies focusing on melatonin, ramelteon, tasimelteon, or agomelatine in the context of insomnia or circadian rhythm disorders. Exclusion criteria: preclinical or animal-only studies, articles without original data or peer-review, studies focusing on unrelated indications, studies not reporting sleep-related outcomes. Relevant data were extracted and synthesized narratively, with emphasis placed on pharmacokinetics, receptor selectivity, and clinical outcomes. The quality and validity of included studies were considered during interpretation, although no formal risk of bias tool was applied. Moreover a recent structured narrative review have applied similar methodological strategies in insomnia research, thereby supporting the validity of our approach [24].

## 3. Melatonergic Receptor Neurophysiology

### 3.1. Characterization of MT1 (Initiation) and MT2 (Diurnal Phase) Receptors

M1 and M2 receptors are implicated in circadian rhythm modulation, sleep and mood variation [25]. They are G protein-coupled receptors (GPCR) activated specifically by melatonin that is released from the pineal gland [26]. This molecule is also synthesized in extrapineal tissues such as the gastrointestinal tract (enterochromaffin cells), skin, bone marrow, thymus, and immune cells [27]. There is evidence that melatonin is locally produced in the retina; it is involved in local circadian entrainment but not in systemic release of melatonin [25].

Genetic and expression research reveal that MT1 receptors are present in the suprachiasmatic nucleus (SCN) of the hypothalamus, hippocampus, substantia nigra, cerebellum, central dopaminergic pathways, ventral tegmental area, and nucleus accumbens [28,29]. Expression signals were also detected in the retina, ovary, testis, mammary gland, coronary arteries, gallbladder, liver, kidney, skin, and immune system [28]. It should be taken into consideration that this data is primarily obtained from transcriptomic studies, while direct validation on the protein level is limited due to the lack of high-specificity antibodies. MT2 receptor expression has most frequently been described in the central nervous system, with additional reports in the lung, heart, aorta, myometrium, granulosa cells, immune cells, duodenum, and adipose tissue [28,29].

Both MT1 and MT2 receptors are situated in the SCN, specific brain regions, and peripheral tissues, where they transduce photoperiodic information and modulate physiological processes [25]. Previous studies have shown that the MT1 receptor is responsible for the initiation of melatonin’s effects, while the MT2 receptor is primarily involved in the modulation of the diurnal phase [30].

It has been established that MT1 and MT2 receptors can form heterodimers with the serotonin receptor 5-HT_2C_. This is of particular importance in understanding the antidepressant mechanism of agomelatine. The formation of MT2/5-HT_2C_ dimers results in greater efficacy than MT1/5-HT_2C_ heterodimers and 5-HT_2C_ homodimers. In addition, MT1 and MT2 receptors can combine different signal transduction cascades, resulting in a unique cellular response. Receptor sensitivity to specific signals changes over the 24 h diurnal cycle. This relationship can be modulated by melatonin itself, i.e., homologously, as well as heterologously, due to other signals, such as light or estrogen effects [27,31,32,33].

Pharmacologically utilized clinical medicines were complemented with augmenting approaches, and one such research using experimental ligands disassembled the molecular-level operation of melatonin receptors. Saturation binding with [^3^H]-melatonin, for example, was capable of identifying high- and low-affinity binding states for the G-protein-coupled and uncoupled receptor conformations of MT1 and MT2 receptors [34]. High-resolution structural studies—such as cryo-EM of MT1 and MT2 in the presence of ligands such as ramelteon or 2-iodomelatonin—have revealed orthosteric binding pocket structure and subtype selectivity mechanism [35]. Receptor structure modeling and virtual screening have shown new chemotypes of sub-micromolar potency with biased G_i_ in contrast to arrestin signaling profiles [36]. In addition, quantum-mechanical docking and dynamics simulations have identified key amino acid interactions (e.g., Gln181/Gln194, Phe179/Phe192) that participate in ligand binding affinity and receptor activation. Such mechanistic insights from non-therapeutic ligands feed into the knowledge of receptor conformational states, subtype selectivity, and signal bias that can inform future drug design [37].

### 3.2. Long-Term Potentiation Variability

**Figure 2:**
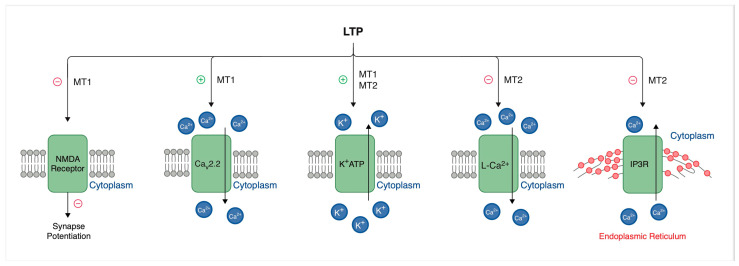
Effects of MT1 and MT2 activation on the modulation of long-term potentiation; MT—melatonin; MT1—melatonin receptor 1; MT2—melatonin receptor 2; NMDA receptor—N-methyl-D-asparate receptor; Ca_v_2.2—N-type calcium channels; K^+^ATP—ATP-sensitive potassium channel; L-Ca^2+^—L-type calcium channel; IP3R—inositol 1,4,5-trisphosphate receptor.

Melatonin, acting through its MT1 and MT2 receptors, can modulate long-term potentiation (LTP) in a manner that depends on receptor type, location, and physiological conditions (Figure 2). The MT1 receptor typically inhibits LTP, while MT2 can have both inhibitory and enhancing effects. In the hippocampus, melatonin plays a significant role in modulating NMDA receptor activity and regulating cyclic adenosine monophosphate (cAMP) levels, which in turn influences the efficiency of LTP. Furthermore, melatonin possesses antioxidant properties that may protect neurons from oxidative stress, thereby potentially impacting synaptic plasticity [38].

For instance, the MT1 receptor exerts predominantly inhibitory effects on long-term potentiation. Stimulation of the MT1 receptor leads to decreased level of cAMP through the inhibition of adenylate cyclase (AC). The following decrease leads to the reduced activation of protein kinase A, a key enzyme in the induction of LTP. In addition, stimulation of the MT1 receptor reduces NMDA receptor activity, further reducing synaptic potentiation. These inhibitory effects are particularly important in the hippocampus, where melatonin’s role in attenuating excitatory neurotransmission contributes to the regulation of circadian cycles and memory formation [39].

Prior research suggests that the MT2 receptor has multiple effects on long-term potentiation, exerting either facilitatory or inhibitory effects depending on the neuronal microenvironment. Activation of the MT2 receptor has been shown to promote LTP by modulating intracellular calcium signaling, which is critical for synaptic potentiation. Contrarily, MT2 receptor activation can also enhance inhibitory neurotransmission through GABAergic pathways, which results in suppression of LTP. This dual functionality suggests that MT2 receptors play a varied role in maintaining the balance between excitatory and inhibitory inputs within neural circuits [40]. These melatonin-mediated effects on LTP are of immediate relevance to sleep-dependent memory consolidation. Of particular interest is that melatonin has been reported to modulate hippocampal LTP in a time-of-day–dependent fashion [41] and potentially rescue sleep-deprivation-impaired synaptic plasticity [42]. Also interesting is that agomelatine improves memory performance and restores BDNF and CREB expression in stress-exposed mice [43].

### 3.3. Intracellular Pathways, Modulation of Neuronal Activity of Suprachiasmatic Nucleus and Other CNS Areas (Hypothalamus, Cortex, Hippocampus, N. accumbens)—Effect of Modulation on a Diurnal Cycle

#### 3.3.1. MT1 Receptor Signaling Pathway

**Figure 3:**
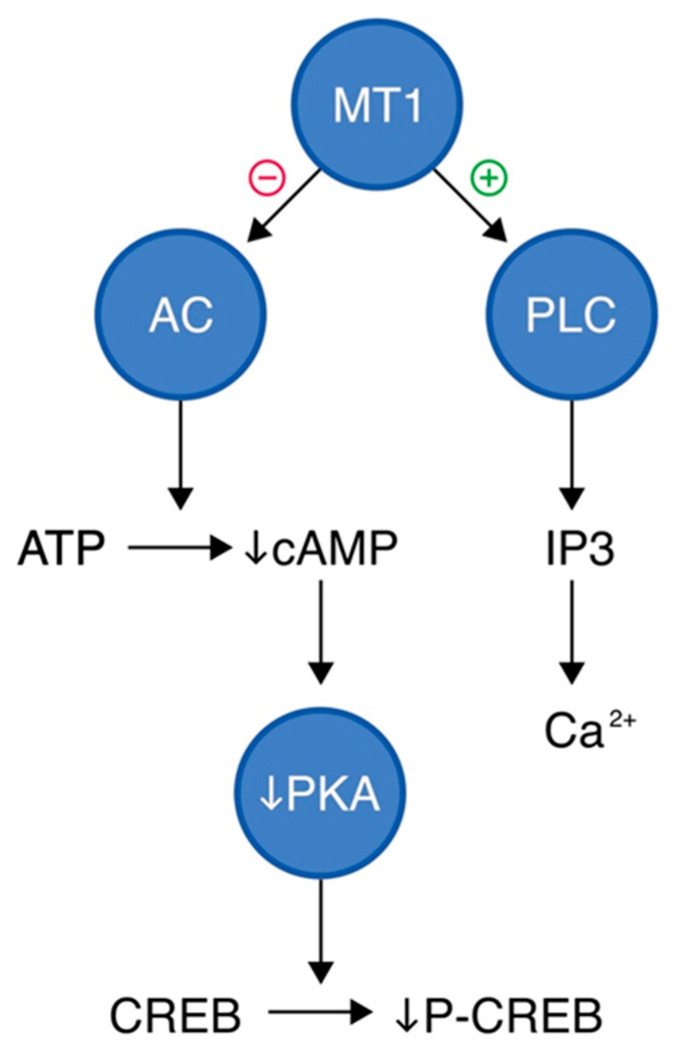
MT1 receptor signaling pathway; AC—adenylyl cyclase; PLC—phospholipase C; IP3—inositol 1,4,5-trisphosphate; Ca^2+^—calcium ion; PKA—protein kinase A; CREB—cAMP-response element-binding protein.

Upon melatonin binding, MT1 activates two parallel downstream pathways: inhibition of AC and activation of phospholipase C (PLC), as presented in Figure 3. Activation of the MT1 receptor leads to inhibition of adenylyl cyclase, resulting in reduced conversion of ATP to cAMP. The decreased cAMP levels subsequently lead to reduced activation of protein kinase A (PKA). Since PKA is responsible for phosphorylating transcription factors such as CREB (cAMP response element-binding protein), its inhibition results in decreased levels of phosphorylated CREB (P-CREB). This suppresses the transcription of CREB-regulated genes, thereby modulating cellular responses such as neuronal excitability and circadian rhythm regulation [44,45]. In parallel, MT1 receptor activation can stimulate PLC in certain cellular contexts. PLC activation leads to hydrolysis of phosphatidylinositol 4,5-bisphosphate (PIP_2_) into inositol trisphosphate (IP_3_) and diacylglycerol (DAG), which can elevate intracellular calcium levels and activate protein kinase C (PKC), although these steps are not provided in the diagram. This arm of the signaling cascade may contribute to additional cellular effects, such as modulation of ion channel activity or further gene regulation [45]. Through these pathways, the MT1 receptor plays a central role in the regulation of circadian rhythms, sleep induction, and neuroendocrine signaling. The suppression of CREB phosphorylation particularly impacts gene transcription patterns that are critical for the timing of circadian processes in the SCN of the hypothalamus [46,47].

#### 3.3.2. MT2 Receptor Signaling Pathway

**Figure 4:**
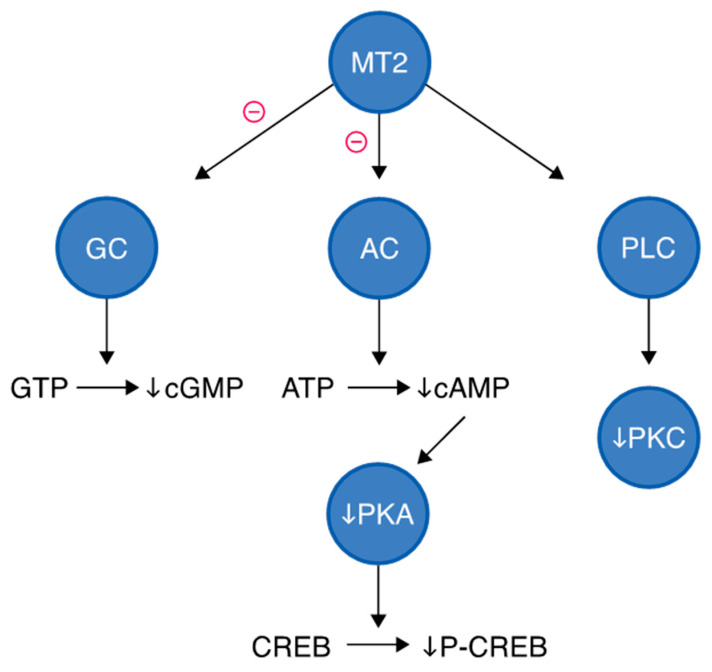
MT2 receptor signaling pathway; GC—guanylyl cyclase; AC—adenylyl cyclase; PLC—phospholipase C; PKC—protein kinase C; PKA—protein kinase A; CREB—cAMP-response element-binding protein.

Activation of MT2 leads to three major signaling pathways, such as inhibition of guanylyl cyclase (GC), inhibition of adenylyl cyclase, and activation of phospholipase C, as presented in Figure 4. Upon melatonin binding, MT2 inhibits adenylate cyclase activity via G_i_ protein coupling, reducing the conversion of ATP to cyclic AMP (cAMP) [45]. This results in decreased activation of protein kinase A (PKA). As a consequence, phosphorylation of CREB (cAMP response element-binding protein) is reduced, leading to diminished transcription of CREB-regulated genes. This pathway is particularly relevant to MT2’s role in circadian phase shifting, especially in the suprachiasmatic nucleus. MT2 activation can also inhibit GC, decreasing levels of cyclic GMP (cGMP) from GTP, which is involved in phototransduction and vascular relaxation, suggesting MT2 plays a role in retinal adaptation and vascular function. MT2 may stimulate PLC, leading to the generation of second messengers such as diacylglycerol and inositol trisphosphate. DAG activates PKC, which is involved in cellular excitability, gene transcription, and hormone secretion. This branch may contribute to phase-shifting of circadian rhythms and neuroendocrine effects [48,49].

#### 3.3.3. Modulation of Neuronal Activity of SCN

Previous studies have emphasized that the SCN exerts regulatory control over the efferent projections from the paraventricular nucleus (PVN) of the hypothalamus, thereby modulating the majority of the circadian functions governed by the autonomic nervous system [50]. This includes the sympathetic pathway, where the preganglionic neurons are situated in the intermediolateral column of the spinal cord and project to the postganglionic neurons in the superior cervical ganglion. Within this pathway, the SCN rhythmically regulates the release of noradrenaline, which in turn promotes the activation of the enzyme arylalkylamine N-acetyltransferase and the subsequent production of melatonin by the pineal gland [51].

Melatonin production can be suppressed by light exposure, particularly in the evening, leading to circadian and sleep disturbances [52]. The most effective modulator of the circadian system is light, but there are other factors, such as feeding schedules, physical activity, and hormones like melatonin, which can also shift the clock [25].

The endogenous circadian rhythms of pineal melatonin synthesis are regulated by intrinsic oscillators within the SCN, which are synchronized with daily and seasonal variations in the environmental light-dark cycle [53].

Endogenous melatonin may provide feedback to SCN, stimulating the MT1 and MT2 receptors to induce phase shifts in local and overt circadian rhythms [25,54,55]. The MT1 receptors predominantly play a role in initiating and sustaining the sleep–wake cycle, whereas the MT2 receptors are responsible for synchronizing the circadian pacemaker to environmental light-dark patterns [56].

### 3.4. Interaction with Serotonergic, Dopaminergic and GABAergic, Orexinergic Systems

#### 3.4.1. Serotonergic System

The serotonergic neurons that arise from the raphe nuclei in the brainstem constitute a significant system that enhances arousal [57].

The regulation of arousal by serotonergic neurons has been associated with both 5HT_2A_ and 5HT_2C_ receptors. Activation of the 5HT_2A_ receptor is expected to promote arousal, a conclusion supported by the sedative effects observed with 5HT_2A_ receptor antagonists like ketanserin [49]. Several 5HT_2A_ receptor antagonists, including eplivanserin, M-100907, and pruvanserin, along with an inverse agonist known as APD125, are currently being investigated as potential hypnotic treatments for insomnia [58,59].

In contrast, the activation of 5HT_2C_ receptors results in increased sedation. This phenomenon is linked to the activation of GABAergic interneurons in the brainstem, which in turn inhibits the activity of neurons that promote wakefulness in both the locus coeruleus and the ventral tegmental area (VTA) [60]. 5HT_2C_ receptor antagonists, including agomelatine, enhance locus coeruleus and VTA activity [60]. This agent’s activity was only noticed at micromolar concentrations, while its melatonergic agonism was sub-nanomolar, and therefore the direct role of the serotonergic pathway in its therapeutic effects is uncertain [60].

#### 3.4.2. GABAergic System

Numerous sedative medications function by amplifying the activity of sleep-inducing GABAergic pathways. Benzodiazepines and barbiturates act on GABA_A_ receptor to promote inhibitory neurotransmission, which aims to reduce neuronal activity [61,62]. For instance, when diazepam binds to GABA_A_ receptors, it increases their affinity for GABA. This makes them prone to activating and inhibiting neuronal firing [61,62]. At the cellular and molecular levels, this enhancement occurs through the potentiation of GABA’s effects on the ionotropic GABA_A_ receptor, which governs chloride channels. The GABA_A_ receptor comprises multiple subunits, and the sedative effects are specifically mediated by receptors that include the α1 subunit [55].

#### 3.4.3. Dopaminergic System

Dopaminergic neurons located in the VTA of the midbrain play a significant role in enhancing wakefulness [63,64]. This wakefulness-enhancing effect of the VTA may be, in part, facilitated by an excitatory link to the locus coeruleus. Additionally, modafinil may stimulate the locus coeruleus by inhibiting dopamine reuptake, which in turn amplifies its impact at the ‘meso-coerulear’ site. It has been suggested that dopaminergic neurons could stimulate the orexinergic system [65].

#### 3.4.4. Orexinergic System

Pharmaceuticals that engage with orexin receptors could present significant therapeutic opportunities: agonists are anticipated to promote alertness, while antagonists are likely to induce sedation [66]. Almorexant and MK-4305 (suvorexant) that are antagonists of the orexin-1 and orexin-2 receptors (OX1R/OX2R), promote sleep in humans [67]. The OX1R and OX2R are G protein–coupled receptors that have 7-transmembrane domains and some resemblance to other neuropeptide receptors [68]. Orexin-A acts via both OX1R and OX2R, whereas orexin-B signals primarily via OX2R. Intracellular pathways mediated by G proteins increase the concentration of intracellular calcium, thereby activating the sodium/calcium exchanger and depolarizing target neurons. The inactivation of G protein–regulated inward rectifier (GIRK) channels is also caused by these cascades. Long-lasting increasing neuronal excitability is produced by increased expression of N-methyl-D-aspartate (NMDA) [68]. Orexin neurons may be influenced by information related to circadian rhythms and the timing of wakefulness may be via the dorsomedial nucleus of the hypothalamus (DMH) [67,69].

#### 3.4.5. Arousal-Controlling Neuronal Network

The regulation of arousal and the transitions among wakefulness, non-rapid eye movement (NREM) sleep, and rapid eye movement (REM) sleep is governed by a sophisticated interplay of various hypothalamic and brainstem nuclei that either promote wakefulness or facilitate sleep. These nuclei have been characterized both anatomically and neurochemically, based on the specific neurotransmitters involved in these processes [57,70,71,72].

**Figure 5:**
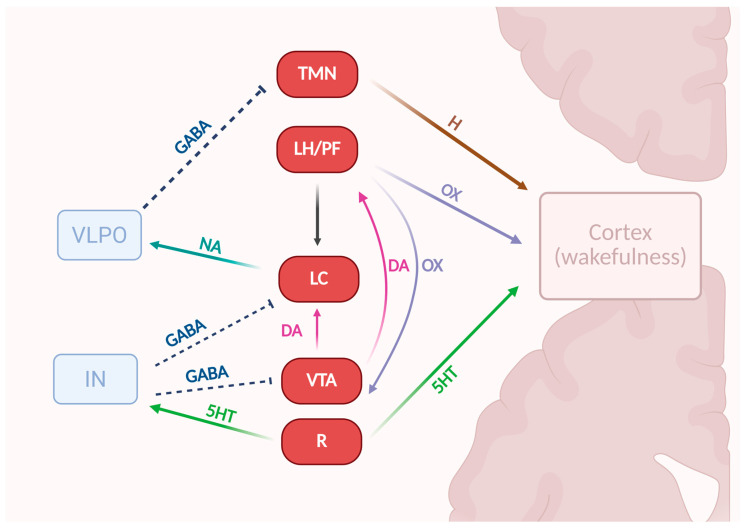
Sleep/arousal neuronal network; Blue boxes—sleep-promoting nuclei. Red boxes—wake-promoting nuclei. LC—locus caeruleus; R—raphe nuclei; TMN—tuberomammillary nucleus; VLPO—ventrolateral preoptic nucleus; IN—GABAergic interneurons; VTA—ventral tegmental area; LH/PF—lateral hypothalamic/perifornical area; Ache, acetylcholine; NA, noradrenaline; H, histamine; Ox, orexine; GABA, γ-aminobutyric acid; DA, dopamine; 5HT, 5-hydroxytryptamine. Receptors: H1, excitatory H1 histamine receptors; 5HT_2A_ and 5HT_2C_, excitatory 5HT receptors [74,75,76].

The wakefulness-promoting nuclei directly activate the cerebral cortex, while the ventrolateral preoptic nucleus (VLPO) encourages sleep by inhibiting the tuberomammillary nucleus (TMN). The locus coeruleus (LC) enhances wakefulness by inhibiting the VLPO. The raphe (R) nucleus contributes to wakefulness by activating the cerebral cortex, though this effect is mitigated by the stimulation of GABAergic interneurons, which inhibit both the LC and the VTA (Figure 5). Lastly, the VTA promotes wakefulness primarily by activating the LC, while the lateral hypothalamus/perifornical area (LH/PF) achieves this mainly through the activation of the TMN and the LC [73,74,75].

## 4. Characteristic of Melatonergic Receptor Agonists

### 4.1. Melatonin

#### 4.1.1. Characteristics of Melatonin

Melatonin is an endogenous hormone with a broad range of effects on the neuroendocrine system and circadian rhythms. It was first isolated in the late 1950s; however, its clinical use in medicine did not become widespread until the 1990s. Exogenous melatonin has demonstrated promising outcomes in the treatment of sleep disorders when administered in pharmacological doses [77,78].

Melatonin’s effect is mediated by interaction with three receptor forms: the G protein-linked MT1 receptor, primarily involved in regulation of the sleep–wake cycle, in particular the REM stage; the GPCR-type MT2 receptor, influencing the NREM sleep stage. The so-called MT3 binding site has been identified as quinone reductase 2 (NQO2), a cytosolic enzyme involved in detoxification and oxidative stress processes. This accounted for the non-GPCR nature of MT3 and suggested that its pharmacological relevance is distinct from MT1/MT2 [79].

#### 4.1.2. Pharmacokinetics of Melatonin

The therapeutic benefits of exogenous melatonin are primarily determined by its bioavailability, which depends on the route of administration, dosage, individual absorption capacity, and hepatic metabolic rate. Orally administered melatonin undergoes extensive hepatic metabolism, with minimal contributions from renal and intestinal pathways. The primary metabolic route involves C-6 hydroxylation, mediated by cytochrome P450 enzymes: CYP1A1, CYP1A2, CYP2C19, and CYP1B1 [80,81,82,83]. Due to the high metabolic activity of liver and associated oxidative stress, melatonin has been shown to exert hepatoprotective effects through its antioxidant properties, mitochondrial protection, and modulation of pro-inflammatory signaling pathways [84].

The impact of endogenous and exogenous melatonin as a function of age was investigated in a study by Zhdanova et al. [85]. The results demonstrated that in the younger age group (mean age: 29 years), peak endogenous melatonin concentrations were significantly higher (100.9 ± 48.6 pg/mL) compared to the older group (mean age: 60 years), which exhibited lower peak levels (34.5 ± 15.4 pg/mL). Following the administration of 0.3 mg of exogenous melatonin, older individuals reached higher and more variable serum melatonin concentrations (254.9 ± 145.7 pg/mL) than their younger counterparts (170.2 ± 22.0 pg/mL). This effect may be attributed to an age-related decline in hepatic enzymatic activity responsible for melatonin metabolism. The influence of sex on melatonin metabolism and bioavailability has not been confirmed in existing studies [86,87].

Oral tablets represent the most common form of melatonin administration, though they are not necessarily the most effective [88,89]. Variability in oral melatonin bioavailability is influenced by factors such as gut microbiome composition, gastrointestinal absorption differences, and genetic polymorphisms of melatonin-metabolizing enzymes. As a result, the oral bioavailability of melatonin ranges from approximately 3% to 74% [90].

Alternative routes of administration have been shown to significantly enhance bioavailability. The study conducted by Zetner et al. [91] demonstrated that rectal and vaginal administration of melatonin resulted in bioavailability rates of 36% and 97%, respectively, suggesting these routes may be both safer and more effective than oral delivery. Furthermore, transdermal administration exhibited prolonged pharmacokinetics, maintaining melatonin plasma levels for up to 14 h, which may benefit sustained therapeutic effects. Studies have indicated that extended-release formulations provide superior clinical outcomes compared to immediate-release forms. Regular melatonin achieves peak plasma concentration (T_max_) within approximately 30–50 min and is rapidly eliminated (t1/2 ≈ 40–60 min) [89,92]. In contrast, extended-release melatonin allows for gradual release over several hours, closely mimicking the physiological secretion pattern of endogenous melatonin by the pineal gland [93,94].

#### 4.1.3. Efficacy of Melatonin in Sleep-Related Clinical Trials

One of the most common indications for melatonin use is the treatment of sleep disorders. Exogenous melatonin improves sleep quality through multiple mechanisms, including reducing sleep onset latency, increasing total sleep duration, improving sleep efficiency, and decreasing nocturnal awakenings. Scientific research on the use of exogenous melatonin in sleep disorders shows considerable heterogeneity across populations and outcomes. According to the American Academy of Sleep Medicine guidelines, melatonin is not recommended as a pharmacological treatment for chronic insomnia in adults, with cognitive behavioral therapy considered the first-line intervention. Nevertheless, numerous studies highlight the effectiveness of melatonin and its potential benefits not only in sleep disorders but also in the regulation of circadian rhythms. The following section summarizes key clinical trials and meta-analyses published evaluating the efficacy of melatonin in sleep disorders.

A study conducted by Luthringer et al. [95] evaluated the effects of 2 mg prolonged-release melatonin (PRM) in patients over 55 years of age with diagnosed primary insomnia. Forty participants were enrolled, with 20 receiving PRM and 20 assigned to placebo. The results demonstrated that in the PRM group, sleep onset latency (SOL) was reduced by an average of 9 min compared with placebo. Moreover, subjective assessments using the Leeds Sleep Evaluation Questionnaire (LSEQ) indicated improved sleep quality in patients treated with PRM. Total sleep time showed only a slight increase, with no statistically significant differences between groups. No adverse effects or withdrawal symptoms were observed following discontinuation of therapy [95].

A similar study, but on a much larger group of patients, was conducted by Wade [96]. The study included 791 patients aged 18 to 80 years with chronic insomnia. Following a two-week placebo run-in period, participants received prolonged-release melatonin (PRM) at a dose of 2 mg administered 1–2 h before bedtime for 29 weeks. During the final two weeks, placebo was reintroduced to assess potential withdrawal effects. Melatonin demonstrated particular efficacy in the subgroup of 281 patients aged >65 years. In this population, a significant reduction in sleep latency (SL) of approximately 15 min and an increase in total sleep time (TST) of approximately 7 min compared with placebo were observed after only three weeks of treatment [96]. The results for this subgroup are summarized in Table 1. Furthermore, the incidence of adverse events did not differ significantly between the melatonin and placebo groups.

Melatonin is also used in pediatric populations. In a study by Appleton et al. [97], the efficacy and safety of immediate-release melatonin were evaluated in children with neurodevelopmental disorders and chronic sleep difficulties. The trial included 146 children aged 3 to 15 years, of whom 110 were included in the primary analysis. Participants were randomized to receive melatonin at doses of 0.5 mg, 2 mg, 6 mg, or 12 mg, or placebo, for a duration of 12 weeks. Children treated with melatonin slept on average 23 min longer, and their sleep onset latency was reduced by approximately 45 min [97].

The study by Smits et al. [98] investigated the effect of melatonin on childhood sleep-onset insomnia. Forty children aged 6 to 12 years with chronic sleep-onset insomnia lasting for more than one year were enrolled. Melatonin was administered daily at a dose of 5 mg for a period of 4 weeks. The results demonstrated that melatonin reduced sleep onset latency by more than one hour and increased total sleep time. No serious adverse effects were reported.

Melatonin represents a promising and safer alternative to traditional hypnotics such as benzodiazepines, which are associated with a high risk of dependence. Studies suggest that melatonin may facilitate benzodiazepine withdrawal while maintaining sleep quality. The therapeutic effects of melatonin are particularly evident in older adults, while its efficacy in younger individuals appears less pronounced, warranting further investigation into the underlying mechanisms responsible for this discrepancy [99,100,101].

Beyond sleep regulation, melatonin has shown potential therapeutic benefits in neurological disorders such as Alzheimer’s disease, Parkinson’s disease, and autism spectrum disorder (ASD). Studies have demonstrated improvements in sleep architecture and regulation in patients with neurodegenerative diseases, along with beneficial effects on behavioral and cognitive function [102,103]. In children with ASD, melatonin use resulted in significant improvements in sleep hygiene, reductions in sleep onset latency, and enhanced overall sleep quality [104,105].

Melatonin is widely recognized as a substance with a favorable safety profile. In most clinical trials, no toxicity-related adverse effects have been reported. For example, the study by Seabra [106] evaluating the effects of prolonged melatonin use (10 mg daily for 28 days) found no significant toxicological changes aside from a minor reduction in stage 1 sleep duration. Reported adverse events, including headaches, dizziness, nausea, and daytime drowsiness, occurred at rates comparable to placebo [106,107].

However, the review by Boutin et al. [108] emphasizes that although melatonin is generally considered a safe substance, in practice, there are risks associated with its misuse, particularly in pediatric populations and with long-term administration. In a study by Bishop-Freeman et al. [109], seven cases of death in children aged 2 months to 3 years were analyzed. In each case, postmortem blood testing revealed melatonin concentrations ranging from 3 to 1400 ng/mL, markedly exceeding the physiological levels expected at this age. Although all deaths were classified as being of undetermined cause, the presence of such elevated melatonin concentrations was considered a potentially significant contributing factor.

It is important to note that the clinical literature also reflects certain controversies regarding the excessive publicity of melatonin supplementation, particularly at very high (‘stratospheric’) doses. While low doses (≤2–5 mg) mimic physiological secretion and demonstrate efficacy with a favorable safety profile, many commercially available formulations, especially in the over-the-counter (OTC) market, provide doses several-fold higher than those used in controlled clinical trials. Some studies have argued that such supra-physiological concentrations not only lack clear additional benefits but may also contribute to misinterpretation of melatonin’s therapeutic potential and increase the risk of adverse events, particularly in pediatric and vulnerable populations [108].

### 4.2. Tasimelteon

#### 4.2.1. Characteristics of Tasimelteon

Tasimelteon is the only drug approved by the FDA (2014) and EMA (2015) for the treatment of non-24 h sleep–wake rhythm disorder (Non-24) holding orphan drug status [110]. This novel agonist of the MT1 and MT2 melatonergic receptors exhibits 2.1–4.4 times greater affinity for the MT2 receptor and is sold as 20 mg hard capsules administered by the oral route [111,112]. The compound has garnered the attention of researchers due to its observed agonism toward serotonin 5-HT_2C_ and 5-HT_2B_ receptors. It has been posited that, similar to agomelatine, tasimelteon could potentially be effective in the treatment of depression [113].

#### 4.2.2. Pharmacokinetics of Tasimelteon

Tasimelteon demonstrates elevated first-pass metabolism in the liver and intestines, with CYP1A2 and CYP3A4/5 exhibiting primary responsibility for biotransformation. The effects of CYP1A1, CYP2C9/19, and CYP2D6 are comparatively minor [114,115]. The bioavailability of this drug is determined by these cytochromes, with an absolute value of 38.3%. Importantly, the bioavailability remains in a linear trend at doses ranging from 3 to 300 milligrams [116]. Tasimelteon demonstrates a binding affinity for proteins of 90%, exhibiting a half-life ranging from 1.3 to 3.7 h in healthy individuals. This characteristic confers a notable therapeutic advantage over melatonin, which possesses a comparatively shorter half-life of 20 to 45 min [117]. According to FDA recommendations, this compound should not be taken with a meal. Similarly to ramelteon, co-administration of tasimelteon with a high-fat meal lowers C_max_ (by 44%) and delays T_max_ (by 1.75 h) compared to fasting. Elderly individuals exhibit around twice the exposure to tasimelteon compared with non-elderly subjects [116,117]. The main metabolites of the drug are M9, M11, M12, M13 and M14, which, due to their approximately 13-fold lower activity toward melatonin receptors, do not affect agent’s efficacy. These metabolites, as well as tasimelteon itself, have no significant affinity for 160 other clinically relevant receptors. Studies indicate that 1% of tasimelteon is excreted in its native form [111,115].

#### 4.2.3. Clinical Efficacy

##### Non-24-Hour Sleep–Wake Disorder

This condition is observed in approximately 50% of individuals with visual impairment. The underlying mechanism pertains to the absence of light perception, which hinders the synchronization of the SCN with the solar cycle [110]. Blindness causes a gradual delay in the diurnal cycle each day, exacerbating nighttime insomnia and daytime sleepiness. Treatment with tasimelteon alleviates these symptoms by resynchronizing the day-night cycle, as well as improving sleep and wakefulness functions [102]. A randomized withdrawal study of the Safety and Efficacy of Tasimelteon (RESET) in the totally blind by Lockley et al. [118] demonstrated that continued treatment with tasimelteon maintained diurnal synchrony in 90% of subjects, while synchrony was maintained in 20% of subjects after discontinuation. The study further confirmed that the compound was safe and well-tolerated, which formed the basis for its approval by the FDA and EMA [118]. It has been posited that tasimelteon may demonstrate higher efficacy in the treatment of Non-24 than ramelteon. Furthermore, it can be used by sighted individuals with the condition [111,119].

##### Jet Lag and Insomnia After Sleep-Time Shift

Polymeropoulos et al. [120] conducted a comprehensive twelve-center, randomized, double-blind study on the treatment of jet lag disorder symptoms with 20 mg of tasimelteon. They demonstrated the efficacy of this drug by simulating trans-Meridian eastward travel with the intention of shifting the diurnal cycle by 8 h through the use of tasimelteon. The use of this drug improved alertness, reduced drowsiness, and shortened sleep latency by −15.1 min [120].

Tasimelteon is effective in treating insomnia after sleep-time shift. Rajaratnam et al. [121] conducted two randomized controlled multicenter trials consisting of Phase II and Phase III. Phase II (*n* = 39) evaluated several drug doses (10–100 mg) following a 5 h shift sleep, assessing sleep efficiency and melatonin rhythm. In Phase III (*n* = 411), the impact of varying doses of 20 to 100 milligrams was compared with placebo, and the latency to sustained sleep (LPS) was measured by polysomnography [121].

In the Phase II study, tasimelteon decreased sleep latency and increased sleep efficiency compared to placebo. Dose-dependent advancement of plasma melatonin rhythm to an earlier time was obtained. The Phase III study demonstrated the efficacy of tasimelteon in reducing the time required to initiate sleep, enhancing sleep efficiency, and decreasing the duration of awakenings subsequent to sleep onset. The drug’s safety profile demonstrated comparable outcomes to those observed in the placebo group [121].

##### Safety and Tolerance of Tasimelteon

The drug is considered highly safe and well-tolerated in both oral and intravenous forms [111,122]. Taking tasimelteon of a dose of 20 mg at bedtime, after 9 h the next day, did not produce clinically significant impairment of driving performance. In contrast to ramelteon, the drug lacks long-acting, active metabolites capable of impacting cognitive function, psychomotor performance, or driver memory [123].

Torres et al. [123] conducted studies suggesting that a single dose of tasimelteon does not undergo clinically relevant pharmacokinetic changes in subjects with mild to moderate hepatic impairment or severe renal impairment, including those on dialysis. Two prospective, single-center studies have demonstrated that there is no necessity to individualize the dosage of subjects with the aforementioned disorders relative to a healthy control population [123].

Bonacci et al. [117] presented in their publication relevant side effects such as headache (15.4% of subjects), elevated ALT (9.6% of subjects), upper respiratory and urinary tract infection (both 5.8% of subjects), somnolence and sleep disorder, and abnormal dreams (5.8%, 5.8%, 7.7%, respectively) [117]. A similar trend was presented in a paper by Nishimon et al. [111], confirming the side effects presented by the manufacturer on the patient information leaflet [111]. Zuo et al. [124] documented a series of new side effects that had not previously been described by the manufacturer. These included insomnia and sleep disorders [124].

### 4.3. Ramelteon

#### 4.3.1. Characteristics of Ramelteon

In 2005, ramelteon was approved by the U.S. Food and Drug Administration (FDA) as the first melatonin receptor agonist targeting MT1 and MT2 receptors for the treatment of insomnia, particularly in patients with difficulty initiating sleep [101].

Ramelteon is a synthetic melatonin receptor agonist that selectively activates the MT1 receptor, thereby facilitating sleep by inhibiting the activity of neurons in the SCN. Additionally, it activates the MT2 receptor, which is responsible for synchronizing the diurnal rhythm [125].

In comparison with melatonin, ramelteon exhibits a six-fold higher affinity for the MT1 receptor and a four-fold higher affinity for the MT2 receptor [126]. At the same time, its affinity for the MT1 receptor is approximately ten times higher than for the MT2 receptor [127]. Ramelteon shows negligible affinity for the MT3 receptor, which is not a classical metabotropic receptor but has been identified as a quinone reductase type 2 (NQO2) receptor, and no significant effect on other receptors [104].

The high selectivity of ramelteon minimizes the risk of dependence and contributes to a favorable safety profile, representing a significant advantage over classical melatonin agonists and traditional hypnotics such as benzodiazepines, which act non-selectively through modulation of GABA_A_ receptors [128].

#### 4.3.2. Pharmacokinetics of Ramelteon

Ramelteon is well absorbed following oral administration; however, its mean systemic bioavailability is approximately 1.8%, primarily due to extensive first-pass hepatic metabolism. After oral dosing, ramelteon typically reaches peak plasma concentrations within approximately 45 min. Its elimination half-life is longer than that of melatonin and ranges from about 0.8 to 1.9 h, depending on the oral dose administered (ranging from 4 to 64 mg) [126,129,130].

The liver plays a central role in the metabolism of ramelteon, with the cytochrome P450 isoenzyme CYP1A2 being primarily responsible for its oxidation. The predominant metabolite, M-II, reaches higher plasma concentrations than the parent compound and exhibits a longer half-life, ranging from 2 to 5 h. Although M-II demonstrates affinity for MT1 and MT2 receptors, its pharmacological activity is significantly lower compared to that of ramelteon [131,132,133].

#### 4.3.3. Safety and Tolerance of Ramelteon

In a systematic review and meta-analysis conducted by Kuriyama et al. [134], the efficacy and safety profile of ramelteon in the treatment of insomnia in adults were evaluated. The analysis included 13 randomized, placebo-controlled trials involving a total of 5812 patients diagnosed with primary, chronic, or psychophysiological insomnia. Most participants received 8 mg of ramelteon orally before bedtime. Both subjective and objective sleep measures were used to assess treatment efficacy, including polysomnographic evaluations [134].

The meta-analysis demonstrated a statistically significant improvement in subjective sleep latency (sSL), along with a reduction in LPS. Sleep efficiency (SE) also showed a moderate improvement, and a slight increase in total sleep time (TST) of 7.26 min was observed. Sleep quality, assessed using a 7-point Likert scale, improved modestly. However, there was no statistically significant difference in subjectively assessed total sleep time (sTST) between the ramelteon and placebo groups.

The findings support the favorable safety profile and good tolerability of ramelteon, with somnolence being the only notable adverse effect. The authors highlight the need for long-term studies, as the clinical effect of ramelteon appears to be modest over relatively short treatment durations [134].

In a meta-analysis conducted by Maruani et al. [135], the efficacy of ramelteon for the treatment of chronic insomnia (as defined by DSM-IV/ICSD criteria) in adults without comorbid medical conditions was evaluated. The analysis included 1804 patients treated with ramelteon and a corresponding placebo group. The dosing range of ramelteon in the included clinical trials varied from 4 mg to 16 mg per day; however, the standard regimen consisted of 8 mg orally, administered once daily approximately 30 min before bedtime [135].

After four weeks of treatment, significant improvements were observed in several sleep parameters among patients receiving ramelteon. These included reductions in both objective sleep onset latency (oSOL) and subjective sleep onset latency (sSOL). Additionally, data obtained from polysomnography and actigraphy showed increases in both objective total sleep time (oTST) and subjective total sleep time (sTST). Patients undergoing long-term treatment with ramelteon (>4 weeks) also experienced meaningful therapeutic benefits compared to placebo. During extended treatment, oTST increased by 2.02 min, and sTST increased by 14.5 min. Although the magnitude of improvement in sleep parameters was smaller in the long-term treatment group compared to short-term users, the therapeutic benefits of ramelteon were sustained over time, accompanied by a very favorable safety profile and good overall tolerability [135]. The effectiveness of Ramelteon in clinical trials is presented in the Table 2.

A melatonin receptor agonist such as ramelteon demonstrates a favorable safety profile and good tolerability in the treatment of insomnia, particularly in adult and older populations (≥50 years). Findings from both short-term and long-term studies have shown statistically significant improvements in sleep parameters, including total sleep time and sleep latency, as measured by both subjective and objective sleep assessments.

While the meta-analysis by Kuriyama et al. [134] characterized the therapeutic effect of ramelteon as moderate, more recent studies [130,131] suggest more pronounced therapeutic benefits, especially in older patients who use ramelteon for periods longer than four weeks. Notably, these improvements in sleep parameters are sustained during long-term use without an increased risk of adverse effects.

Given the discrepancies in study findings, there is a clear need for well-designed, long-term clinical trials to further validate the potential benefits of ramelteon in patients with insomnia.

### 4.4. Agomelatine

#### 4.4.1. Characteristics of Agomelatine

In February 2009, the European Medicines Agency (EMA) approved agomelatine, an antidepressant with sleep-modulating properties, for the treatment of major depressive episodes in adults [136].

Agomelatine is a novel, atypical antidepressant classified within the “MASS” group—melatonin receptor agonists and selective serotonin receptor antagonists. The drug exerts its effects by activating melatonin MT1, MT2 receptors and antagonizing serotonin 5-HT_2C_, 5-HT_2B_ receptors [137]. Importantly, this antagonistic activity has been demonstrated only at micromolar concentrations, whereas melatonergic agonism occurs in the sub-nanomolar range, which makes the actual contribution of the serotonergic pathway to its clinical profile uncertain [60].

Through its antagonism of serotonin receptors, agomelatine increases the levels of norepinephrine and dopamine in the prefrontal cortex, contributing to its antidepressant properties and cognitive-enhancing effects. Additionally, by modulating melatonergic receptors, agomelatine helps restore normal circadian rhythms, which positively influences sleep regulation [51].

#### 4.4.2. Pharmacokinetics of Agomelatine

Following oral administration, agomelatine is rapidly and extensively absorbed; however, its absolute bioavailability is low (less than 5% at therapeutic doses) and shows considerable interindividual variability. Peak plasma concentrations are typically reached within 1 to 2 h. Agomelatine is highly bound to plasma proteins, with approximately 95% bound.

The drug undergoes extensive hepatic metabolism, primarily via cytochrome P450 isoenzymes CYP1A2, CYP2C9, and CYP2C19. Its metabolites, including hydroxylated and demethylated derivatives, lack pharmacological activity and are rapidly conjugated and excreted. The mean plasma elimination half-life of agomelatine ranges from 1 to 2 h. The drug is predominantly excreted via the urine (approximately 80%) in the form of metabolites [138].

#### 4.4.3. Safety and Tolerance of Agomelatine

Lemoine et al. [139] conducted an evaluation of subjective sleep quality in patients treated with agomelatine compared to those receiving venlafaxine, a serotonin-norepinephrine reuptake inhibitor (SNRI). In this 6-week randomized, double-blind trial, 332 patients diagnosed with major depressive disorder (MDD) according to DSM-IV criteria were enrolled. The study compared the efficacy of agomelatine (25–50 mg/day) and venlafaxine (75–150 mg/day), with dose adjustments permitted after two weeks of treatment. Subjective sleep quality was assessed using the Leeds Sleep Evaluation Questionnaire (LSEQ) [139].

The results demonstrated that agomelatine significantly improved the “getting to sleep” parameter on the LSEQ, with a mean score of 70.5 ± 16.8 mm, compared to 64.1 ± 18.2 mm for venlafaxine. The between-group difference was 6.36 mm and reached statistical significance (*p* = 0.001), with beneficial effects observed as early as the first week of therapy. Significant improvements were also noted in several secondary sleep parameters, including sleep quality (*p* = 0.021), night-time awakenings (*p* = 0.040), behavior following awakening (*p* = 0.024), and the cumulative insomnia score from the HAM-D scale (items 4, 5, and 6; *p* = 0.044). Furthermore, global improvement assessed using the Clinical Global Impression (CGI) scale favored agomelatine (*p* = 0.016). Agomelatine demonstrated comparable antidepressant efficacy to venlafaxine, while providing superior benefits in terms of the speed and extent of improvement in subjective sleep quality among patients with unipolar depression [139].

The international, multicenter, randomized, double-blind study conducted by Quera-Salva et al. [140] aimed to compare the effects of agomelatine (25–50 mg/day) and escitalopram (10–20 mg/day) on sleep parameters assessed by polysomnography in patients with major depressive disorder treated over a period of up to 24 weeks. A total of 138 outpatients participated in the study, with 71 receiving agomelatine and 67 receiving escitalopram. By the second week of treatment, patients in the agomelatine group demonstrated a reduction in sleep latency, with statistically significant differences observed between the two groups at all time points. Escitalopram was associated with a prolongation of REM sleep latency compared to agomelatine, a difference that was statistically significant at each study visit. Furthermore, agomelatine preserved the number of sleep cycles, whereas escitalopram reduced them, with significant between-group differences noted throughout the study duration. Assessments using visual analog scales revealed that patients treated with agomelatine reported better morning condition and less daytime sleepiness compared to those treated with escitalopram [140].

Hsing et al. [141] investigated the association between the use of agomelatine and other antidepressants and the need for concomitant use of sedative-hypnotic medications. The study included 7961 patients, of whom 1985 were treated exclusively with agomelatine, while 5976 received other antidepressants. A total of 3322 individuals in the cohort used sedative-hypnotic medications. Among them, 811 patients (40.86%) were in the agomelatine-only group, and 2511 patients (42.02%) were in the group receiving other antidepressants. After adjusting for potential confounders, the odds ratio (OR) for the use of sedative-hypnotics in the agomelatine-only group was 0.892 (95% confidence interval [CI]: 0.306–1.601; *p* = 0.533) compared to the control group. The relative risk (RR) of sedative-hypnotic use in the same group was 0.910 (95% CI: 0.312–1.633; *p* = 0.520) relative to the control group. These findings indicate that patients treated exclusively with agomelatine did not exhibit an increased need for sedative-hypnotic medications compared to those receiving other antidepressants [141].

In an off-label treatment setting, Grosshans et al. [142] administered agomelatine at doses ranging from 25 to 50 mg nightly to nine patients diagnosed with alcohol dependence and chronic sleep disturbances. Sleep quality was assessed using the Pittsburgh Sleep Quality Index (PSQI) both prior to the initiation of agomelatine therapy (T1) and after six weeks of treatment (T2). At baseline, all participants had a global PSQI score above 10 (mean [SD] = 13.1 [1.7]), indicating severe insomnia. Following six weeks of agomelatine treatment, a statistically significant reduction in the global PSQI score was observed in all cases, with a mean post-treatment score of 7.8 (SD = 1.7) (t = 12.8; *p* = 0.00) [142]. The efficacy of agomelatine in clinical sleep studies is presented in Table 3.

Findings from the international, prospective, non-interventional observational study by Gorwood et al. [143] confirm the favorable safety profile of agomelatine when used in accordance with recommended dosing guidelines under conditions of routine clinical care [143].

In summary, agomelatine has proven to be an effective and well-tolerated treatment for insomnia, particularly in patients with comorbid depression. Its unique mechanism of action, involving modulation of both the melatonergic and serotonergic systems, facilitates the restoration of normal circadian rhythms and improves sleep quality. Clinical studies have shown that agomelatine not only enhances sleep parameters—such as sleep onset latency, number of awakenings, and subjectively perceived sleep quality—but also does not increase the need for additional hypnotic medications when compared to other antidepressants. Moreover, therapeutic benefits have been observed as early as the first weeks of treatment, highlighting the rapid onset of action. Agomelatine thus represents a safe and promising therapeutic alternative for the management of sleep disturbances, especially when associated with depressive disorders.

## 5. Herbal Medicines

Conventional insomnia therapies, such as synthetic antidepressants and anxiolytics, may cause side effects including headaches, sexual dysfunction, addiction, seizures, and suicidal thoughts or behaviors. Furthermore, most patients develop a tolerance to these drugs, leading to significantly higher consumption of sleeping pills and anxiolytic agents [144,145].

However, due to concerns about the long-term use of these treatments, there is a growing interest in multi-targeted approaches to treating sleep disorders. These approaches include traditional, complementary, and integrative medicines (TCIM), such as medicinal plants that promote sleep. TCIM offers a cost-effective and less hazardous alternative, especially for individuals with chronic conditions. Medicinal plants are popular worldwide due to their cost-effectiveness, ease of access, and fewer side effects compared to benzodiazepines [146]. Medicinal herbs are believed to contain effective agents for treating insomnia and other sleep disorders. International organizations, such as the World Health Organization (WHO), are making greater efforts to develop and promote the quality of these products. Additionally, non-pharmaceutical therapies are highly recommended as initial treatments for mild to moderate conditions and symptoms, such as non-severe insomnia, particularly for the elderly. Medicinal plants are widely used because they are easily accessible and affordable. They are also generally considered safer than modern medicine [147].

This subsection explores the potential benefits of utilizing plant species in the treatment of insomnia. A list of selected medicinal plants is provided in Table 4.

### 5.1. Matricaria chamomilla L. (Chamomile); Asteraceae

*Matricaria chamomilla* (Asteraceae) is an annual flowering plant native to Europe and Asia. Thanks to its calming and sleep-promoting properties, it has a long history of use in traditional medicine. It shows promise in treating neurological conditions such as generalized anxiety disorder and comorbid depression [148]. One randomized, double-blind, placebo-controlled study demonstrated a significant reduction in mean anxiety symptoms (*p* = 0.047). An additional exploratory study revealed substantial decreases in total and core depression scores (*p* < 0.05) [149]. The plant’s efficacy is attributed to its complex phytochemical profile, which includes terpenoids (e.g., α-bisabolol and chamazulene) and phenolic metabolites (e.g., phenolic acids, flavonoids [e.g., apigenin and luteolin], and coumarins). These metabolites are believed to contribute to the plant’s sedative properties. They have a synergistic effect that enhances chamomile’s clinical utility [150]. The mechanisms underlying the sedative effects of German chamomile are not fully understood, but they may involve modulation of GABA receptors. In vitro studies have shown that apigenin, a major flavonoid in chamomile, can bind to GABA receptors and enhance their activity. This leads to increased neuronal inhibition and relaxation. Additionally, chamomile extracts have been reported to modulate serotonin and dopamine receptors, which may contribute to their anxiolytic and mood-enhancing effects [151].

A randomized, double-blind, placebo-controlled trial examined the impact of chamomile extract on sleep quality and fatigue in 60 older adults residing in nursing homes. After four weeks of treatment, the study revealed notable enhancements in sleep quality and fatigue scores among the chamomile group versus the placebo group. The authors concluded that chamomile could be a safe and effective alternative to conventional sleep medications for older adults [152]. A study investigating the effects of chamomile (1500 mg/day) on subjects with generalized anxiety disorder (GAD) and comorbid depression found significant reductions in Hamilton Rating Scale for Depression (HRSD) core symptom scores (*p* < 0.023), as well as a trend toward reductions in HRSD total scores (*p* = 0.14) and Beck Depression Inventory (BDI) total scores (*p* = 0.060), particularly in subjects with comorbid depression. These results suggest that the extract may possess clinically significant antidepressant properties alongside its anxiolytic activity [153]. Additionally, chamomile is a potent remedy for physical and psychological discomfort in patients with depression. Chamomile tea made from flower heads can effectively alleviate depressive symptoms and improve sleep quality in postpartum women [154], which was confirmed in a randomized, double-blind, placebo-controlled trial that examined the effects of chamomile tea on sleep quality and depression in 80 postpartum women. The study reported significant improvements in sleep quality and depression scores in the chamomile group compared with the placebo group after two weeks of treatment [154].

*Matricaria chamomilla* is considered safe. According to a systematic review of 69 clinical trials, the most common side effects were mild and temporary. These included gastrointestinal complaints, dizziness, and allergic reactions [150]. However, chamomile may interact with certain medications. This applies in particular to medicines that are metabolized by cytochrome P450 enzymes. Individuals with allergies to plants in the Asteraceae family (e.g., ragweed or chrysanthemums) should use chamomile cautiously, because cross-reactivity may occur [155]. Further research is needed to determine the most effective dosage, duration, and population for using chamomile to treat insomnia [16].

### 5.2. Melissa officinalis L. (Lemon Balm)

Melissa officinalis L. (MO; lemon balm) (Lamiaceae) is native to the Mediterranean region and western Asia. It contains bioactive volatile compounds, triterpenes, phenolic acids, and flavonoids [159], determining also a beneficial clinical effect on anxiety-related insomnia. The class includes cinnamic acid, coumarin acids, ferulic acids, chlorogenic acid, and, mostly, rosmarinic acid [164].

Among the numerous botanicals that have been studied for their psychopharmacological effects, Melissa officinalis leaf extract has emerged as a promising agent for calming the central nervous system (CNS) and improving mood. Its efficacy was evaluated in vitro by measuring its ability to inhibit GABA-T and monoamine oxidase A (MAO-A) in hydrogen peroxide (H_2_O_2_)-exposed SH-SY5Y cells using a standardized, phospholipid-carrier-based lemon balm extract versus an unformulated, dry lemon balm extract. MO extract supplementation may help ameliorate emotional distress and related conditions [160].

Akhondzadeh et al. [161] conducted a double-blind study evaluating the effect of an MO extract with a fixed dose of 60 drops per day in patients aged 65 to 80 years with mild to moderate memory loss (*n* = 42; 18 women and 24 men). The results demonstrated significant cognitive improvement, with better outcomes on the ADAS-cog and CDR-SB scales reaching statistical significance (ADAS-cog: d.f. = 1, F = 6.93, *p* = 0.01; CDR: d.f. = 1, F = 16.87, *p* < 0.0001). The frequency of agitation decreased as well, suggesting that the extract is valuable in managing mild to moderate memory loss and reducing agitation in these patients. Further studies indicate its potential to improve mood, as evidenced by gains in Depression, Anxiety, and Stress Scale (DASS) scores. A 600 mg dose of M. officinalis improved the negative emotional effects of the Defined Intensity Stressor Simulation (DISS), leading to increased self-ratings of calmness and decreased self-ratings of alertness (*p* < 0.01). Additionally, a significant increase in the speed of mathematical processing was observed with no reduction in accuracy after ingestion of the 300 mg dose [162,163].

Study Pierro et. al [156] assessed the effect of lemon balm on sleep quality in thirty participants (13 men and 17 women). The study assessed the following parameters: total sleep duration, light sleep, slow-wave sleep (SWS), and (iv) REM sleep, as well as the distribution of sleep stages. Improvements in sleep quality parameters were observed. Statistically significant differences (*p* < 0.05) were found in deep sleep and REM sleep, with an increase in time spent in deep sleep and a decrease in time spent in REM sleep in the lemon balm group. The authors observed no significant changes in the following parameters: light sleep time, wakefulness time, or total sleep time. The study did not reveal any significant changes in the perceived level of anxiety. These results indicate that both sleep quality and subjective sleep perception significantly improved after administration of lemon balm compared to placebo. The different study [158] observed an increase in slow-wave sleep (SWS) in people taking lemon balm. Although there is disagreement in the literature regarding the actual role of SWS and REM sleep, authors [158] demonstrated a correlation with improved daytime performance, motor speed, and executive function, as well as improved perception of sleep quality and a sense of nocturnal restfulness. These results suggest that slow-wave sleep may be crucial for sleep quality and, consequently, optimal daytime performance [158]. Another study with 30 participants confirmed significant improvements in sleep quality and reduced insomnia severity, as evidenced by increased deep and REM sleep duration in the study participants [156]. Consistent with data on the effects of lemon balm on GABA-T [160], the study found that lemon balm effectively improved sleep quality and reduced insomnia severity in participants. Furthermore, participants reported subjective improvements in sleep quality, suggesting that lemon balm supplementation positively impacted sleep perception.

### 5.3. Nigella sativa L. (Black Cumin)

*Nigella sativa* L. (black cumin) (Ranunculaceae), also known as black cumin or black seed, has garnered scientific interest due to its potential in managing mental health disorders [165]. N. sativa essential oil (NSO) contains several bioactive metabolites, including thymoquinone (TQ), thymohydroquinone, thymol, carvacrol, β-sitosterol, nigellicine, and nigellidine. N. sativa has been associated with memory enhancement, possibly due to its antioxidant and anti-cholinesterase properties. TQ exhibits anti-anxiety effects by modulating gamma-aminobutyric acid (GABA) and nitric oxide (NO) levels in the brain and plasma [166]. TQ also exhibits significant anxiolytic effects under stress by reducing plasma nitrite and brain GABA concentrations [167]. In a randomized, double-blind, placebo-controlled trial, Mohan et al. [168] found that thymoquinone-rich black cumin oil extract (BCO-5) significantly modulated the stress-sleep-immunity axis with no side effects, restoring restful sleep. Seventy percent of participants in the BCO-5 group reported satisfaction with their sleep patterns on day seven, and 79% reported satisfaction on day fourteen. Furthermore, inter- and intra-group analyses of total Pittsburgh Sleep Quality Index scores on days 45 and 90 revealed BCO-5’s efficacy in improving sleep (*p* < 0.05). Furthermore, significant modulation of melatonin, cortisol, and orexin levels was observed [168]. In another study, Sayeed et al. [169] examined the effects of a 500 mg *Nigella sativa* capsule taken twice daily for nine weeks in healthy elderly volunteers. At the end of the study, the volunteers showed improved cognition, memory, and attention. Similarly, a computed tomography scan performed on healthy adolescent males aged 14–17 years revealed modulatory effects on cognition, mood, and anxiety associated with taking a 500 mg N. sativa capsule daily for four weeks [169]. Asadi et al. [170] obtained contrasting results in their study [170] using black cumin (*Nigella sativa*) and zolpidem to assess their effects on improving sleep quality, hot flashes, and heart palpitations in healthy postmenopausal women. The study included 60 participants (twenty in the control group, 20 in the black cumin group, and 20 in the zolpidem group). The first group received 5 mg of zolpidem for 8 weeks, the second group took 600 mg of black cumin for 8 weeks, and the third group received a placebo for 8 weeks. In the group taking black cumin (*Nigella sativa*), Asadi et al. [170] did not observe a significant improvement in sleep quality (*p* = 0.07), hot flashes (*p* = 0.15), or palpitations (*p* = 0.56). In the group of women taking zolpidem, a significant improvement in sleep quality (*p* = 0.01) and a reduction in night sweats (*p* = 0.049) were observed. Based on the conducted study, it was concluded that zolpidem was effective in improving sleep quality in postmenopausal women, while Nigella sativa L. was not effective in alleviating vasomotor symptoms or improving sleep quality [170].

### 5.4. Valerian (Valeriana Officinalis)

Valerian (*Valeriana officinalis*) is a perennial plant native to North America, Asia, and Europe, whose root is believed to have sedative and hypnotic properties. Valerian is one of the medicinal plants used to reduce anxiety and sleep disorders [171]. Valerian contains 150 to 200 different substances, including essential oils, ketones, phenols, iridoid esters, valeric acid, aminobutyric acid, arginine, tyrosine, glutamine, as well as noncyclic, monocyclic, and bicyclic hydrocarbons [172]. The U.S. Food and Drug Administration (FDA) lists valerian as a dietary supplement with no contraindications to its use [171]. Although the exact mechanism of valerian’s action on sleep disorders is unknown, it is believed that the plant inhibits the uptake and stimulates the release of GABA [173], which may explain the primary mechanisms by which valerian improves sleep quality. Valerian is also considered a partial agonist at the 5-hydroxytryptamine 2A receptor, which increases melatonin release [174], which may be another mechanism by which the plant improves sleep quality. Research on the efficacy of valerian in humans has produced mixed results and varied in quality. A study by Ziegler et al. [175] compared the effects of six weeks of treatment with valerian extract (600 mg/day) and oxazepam (10 mg/day) in 202 patients. Both groups reported improved sleep quality, and valerian was at least as effective as oxazepam. The effects of valerian and oxazepam were considered very good by 83% and 73% of patients, respectively. Another study [176] assessed sleep quality, anxiety, and depression in 39 hemodialysis (HD) patients after administration of valerian capsules (530 mg of dried valerian root) and a placebo [176]. Valerian was found to significantly improve sleep quality, anxiety, and depression symptoms in patients. Another study showed that valerian can be used as a sleep supplement [177]. In the US, one study examined the effects of valerian on sleep quality in cancer patients undergoing treatment and observed a reduction in sleep problems and daytime sleepiness after using this herbal remedy [178]. Another study, however, found that taking valerian did not significantly improve sleep quality [179]. The study involved 391 participants who were assigned to one of three groups: kava plus valerian placebo (*n* = 121), valerian plus kava (placebo) (*n* = 135), or double placebo (*n* = 135). Participants receiving placebo experienced reductions in anxiety symptoms and insomnia symptoms. Those receiving kava had similar results in the placebo group compared to those receiving kava. Those receiving valerian and placebo reported similar improvements in sleep. Similar results were seen for 83% of participants who used the study compounds for all 4 weeks. Neither kava nor valerian improved anxiety or insomnia more than placebo. In an internet-based randomized, placebo-controlled trial of kava and valerian for anxiety and insomnia, Oxman et al. [180] found no significant differences in sleep quality between the valerian and placebo groups. Similarly, another study conducted in the US found that valerian extract had no significant effect on alleviating sleep disturbances in people with arthritis [181].

### 5.5. Passiflora incarnata L. (Passionflower)

The passionflower (*Passiflora incarnata*), commonly known as passionflower, is a climbing vine with white, purple-tinged flowers. The aerial parts of the plant, flowers, and fruits are used for medicinal purposes. It is a traditional herbal sedative and anxiolytic, and a popular hypnotic used to treat sleep disorders [182,183]. Passiflora incarnata is a source of alkaloids, phenolic compounds, flavonoids, and cyanogenic glycosides. The primary phytochemicals found in the passionflower are flavonoids (apigenin, luteolin, quercetin, and kaempferol) and flavonoid glycosides (vitexin, isovitexin, orientin, and isoorientin) [182,183]. These compounds modulate neurotransmitter systems, primarily gamma-aminobutyric acid (GABA), serotonin, and the adrenergic system, which are important in regulating mood, anxiety, and stress response. No risks to human health have been demonstrated in connection with the use of Passiflora incarnata [184]. To date, there is little scientific evidence regarding the constituents of passionflower responsible for its putative sedative and anxiolytic effects, nor the mechanism by which the plant affects sleep [185]. Although the effects of passionflower in humans have rarely been studied, two clinical trials have demonstrated its effectiveness in treating anxiety [184,186] and attention-deficit hyperactivity disorder [187]. A positive correlation has been demonstrated between anxiety and sleep disturbances [188], and passionflower may therefore improve sleep in humans as a secondary consequence of its anxiolytic effects. The Ngan and Conduit [189] study was conducted to evaluate the effectiveness of passionflower (*Passiflora incarnata*) herbal tea on human sleep, as measured by sleep diaries verified by polysomnography (PSG). The study was conducted in a double-blind, placebo-controlled, repeated-measures design, with counterbalanced treatment order (passionflower tea, each containing 2 g of dried passionflower leaves, stems, seeds, and flowers) vs. placebo tea (parsley tea bags (each containing 2 g of dried Petroselinum crispum)), separated by a one-week washout period. Forty-one participants (14 men and 27 women) received each treatment for one week, during which they drank a cup of tea and kept a sleep diary for seven days. Ten participants also underwent overnight PSG on the last night of each treatment period. In the study, sleep quality was rated significantly better with passionflower compared to placebo (t(40) = 2.70, *p* < 0.01). The study results suggest that consuming a small dose of passionflower tea provides short-term subjective sleep benefits in healthy adults with mild fluctuations in sleep quality. Among the subjective sleep quality parameters, significant improvements in perceived sleep quality (SQ) were observed in the passionflower group, with an average increase of 5.2% compared to placebo. This suggests that passionflower (*Passiflora incarnata*) may be a viable alternative for treating mild SQ-related complaints [189].

Kim et al. [190] reported that *P. incarnata* extract significantly improved sleep latency and duration in both animals and humans with insomnia [190]. A study by Christoffoli et al. [191] found that oral administration (20 patients) of passionflower was associated with improved sleep quality in patients undergoing dental procedures, reduced anxiety levels, and faster sleep onset compared to placebo. These results suggest that *P. incarnata* may be considered as an alternative treatment for sleep disorders (to midazolam) in the treatment of anxiety in dental professionals [191].

In another study [192] of passionflower in children and adolescents (94 patients) with eating disorders comorbid with anxiety and insomnia, the herb was administered at a dose of 200 mg (1–2 tablets daily). It was often used in combination with selective serotonin reuptake inhibitors (SSRIs) (56.5%), atypical antipsychotics (27.7%), or benzodiazepines (7.4%). Treatment was initiated for symptoms of anxiety (75.5%) or insomnia (28.7%). Of the patients with specific outcome data, 53.3% reported improvement in anxiety symptoms, and 45.4% reported improvement in insomnia. The authors suggest that Passiflora incarnata L. has an excellent safety profile and preliminary efficacy and may therefore represent a promising alternative for patients with mild symptoms or for caregivers hesitant to use conventional pharmacotherapy [192].

### 5.6. Lavandula Angustifolia Mill. (Lavender)

*Lavandula angustifolia* (lavender) is one of the most popular medicinal plants used in aromatherapy, as inhaling its essential oils has been a common traditional treatment for sleep disorders. Lavender is called “ the broom of the brain “ in various oriental traditional medicines. Lavender’s scent has been used as an anxiolytic, anticonvulsant, analgesic, sedative, and sleep-inducing agent [193,194]. The most important components of lavender oil are linalyl acetate and linalool, which have sleep-inducing properties [195,196]. Thanks to the action of α-aminobutyric acid, lavender essential oil induces sleep primarily in the amygdala, acting on the lymphatic system. Additionally, it improves sleep quality by inducing a hypnotic effect and inhibiting the secretion of acetylcholine [197,198].

Lavender suppresses heart stimulation and lowers blood pressure; therefore, it is useful in the treatment of heart acceleration and high blood pressure [199]. Additionally, its most used administration route is the inhalation of its essential oil (i.e., aromatherapy) alone or in combination by massage. Aromatherapy has been reported to reduce stress [200] as well as decrease anxiety and increase sleep quality in cancer patients [201], other haemodialysis patients [202], and colonoscopy patients [203]. Lavender essential oil has been most frequently studied in the literature, with results suggesting a positive health effect [204,205,206,207,208]. A systematic review of the literature on lavender and sleep found a small to moderate beneficial effect of lavender on sleep [209]. Lavender (Lavandula angustifolia) essential oil has sedative and hypnotic properties and a safety profile [197].

A study [197] assessing the effects of lavender (*L. angustifolia*) inhalation and sleep hygiene on sleep quality and quantity compared to sleep hygiene alone in college students with self-reported sleep problems found improved sleep quality in the lavender group after the intervention and at a two-week follow-up. Safe and effective self-care methods, such as lavender (*L. angustifolia*) and sleep hygiene, are effective first-line treatments for sleep problems [197]. According to the authors, the persistent effect of lavender on sleep quality after a two-week follow-up suggests a balancing or long-lasting effect on the sleep cycle, although the exact mechanism of action is unknown [197]. The authors observed no differences between groups in sleep quantity, although both groups reported falling asleep easily and waking up less frequently after the intervention. The results suggest that lavender and sleep hygiene are safe, accessible, and effective interventions for self-reported sleep problems in college students. The authors acknowledge that further research on their effects in other populations and additional studies on the duration of the intervention effects are needed [197].

In a study by Lari et al. [210], the effect of lavender aromatherapy on sleep quality, quality of life, mood, and FBS in patients with type 2 diabetes was assessed. Fifty-two patients participated in the study and were given lavender essential oil (*Lavandula angustifolia*) and sweet almond oil (placebo). This randomized, crossover clinical trial demonstrated significant improvement in sleep quality in the lavender group. Several studies have also demonstrated a positive effect of lavender aromatherapy on sleep disorders. Najafi et al. [198] confirmed the beneficial effect of lavender aroma on sleep quality in 60 patients on hemodialysis, demonstrating improved sleep quality in the lavender aroma intervention group [198]. Further studies by Moeini et al. [211] and Karadag et al. [196] showed that aromatherapy with lavender essential oil improved sleep quality in patients with coronary artery disease admitted to the coronary intensive care unit. Chien et al. [207] also confirmed that lavender aromatherapy effectively improved sleep quality in women aged 45–55 suffering from insomnia [207].

### 5.7. Melatonin-Containing Plants (MCP)

Melatonin has been identified in various plant species, demonstrating its significance also in the plant kingdom. Notably, some of the species reported to contain melatonin include Coffea canephora (coffee), Coffea arabica, Piper nigrum (black pepper), Lycium barbarum (wolfberry), and Brassica nigra (black mustard), among others. Also, melatonin has been identified in Medicago sativa (alfalfa), Chlorella vulgaris (chlorella), and Oryza sativa (rice). The concentrations of melatonin in these plants can vary widely, with seeds often exhibiting the highest levels, significantly exceeding those found in vertebrate tissues. For instance, melatonin levels in coffee beans can reach up to 6800 ng/g, while seeds of Brassica nigra contain approximately 129 ng/g [81,169,212]. Pistachio species are among the plants with the highest melatonin levels [213,214]. However, there is significant variability in melatonin content in pistachio samples [215], which may be due to differences in season or harvest location, extraction procedure, or detection method (e.g., ELISA, LC-MS) [216]. In the case of pistachios, 200 µg (or more) of melatonin per gram of plant material has been reported [215], while in other studies, high melatonin content of 4–5 mg/g of extract was found in extracts from dried pistachio seeds (*Pistacia vera*) [215].

The presence of melatonin in plants is crucial as it plays a significant role in their physiological processes, including growth regulation, stress response, and defense mechanisms against oxidative stress. This is particularly relevant in reproductive organs, where higher melatonin concentrations have been linked to enhanced resilience against environmental stresses, thus promoting species survival [217,218,219,220].

The bioavailability of melatonin from these plant sources to humans is also noteworthy. Studies suggest that dietary intake of melatonin from plant-based foods, such as fruits and beverages like red wine and coffee, can effectively elevate serum melatonin levels in humans. The concentrations observed in these foods may contribute to the modulation of physiological functions, including circadian rhythm regulation and antioxidant defense mechanisms [220,221,222].

Previously it was proven that the plant species with melatonin present in a phytocomplex may exhibit more favourable properties than synthetic melatonin. The research article by Kukula-Koch et al. [212] presents evidence that phytomelatonin (PHT-MLT), derived from natural sources, exhibits superior antiradical and anti-inflammatory properties compared to synthetic melatonin (SNT-MLT). The study reveals that PHT-MLT demonstrates an enhanced inhibitory effect on the COX-2 enzyme, attributed to the presence of additional phytochemicals within the plant matrix, which not only augment its therapeutic potential but also improve bioavailability. Furthermore, PHT-MLT exhibits a markedly higher capacity for neutralizing free radicals in vitro than its synthetic counterpart, which shows limited activity under similar experimental conditions. The combination of PHT-MLT with vitamin C has been shown to produce synergistic antioxidant effects, suggesting a robust protective mechanism against oxidative stress. These findings show the potential of phytomelatonin-rich formulations as a promising alternative in the prevention and management of disorders associated with oxidative damage and inflammation, thereby advancing our understanding of melatonin’s therapeutic applications in clinical settings. The MCP may constitute an important alternative to the supplementation [212].

### 5.8. Comparison of Efficacy and Cost: Melatonergic Drugs vs. Herbal Preparations

In this review, we emphasized the role of both melatonergic receptor agonists and herbal products in the management of insomnia. While both categories affect circadian regulation and sleep architecture, they differ substantially in clinical efficacy, level of evidence, safety, accessibility, and cost-effectiveness.

Synthetic melatonergic drugs, including melatonin, ramelteon, agomelatine, and tasimelteon, exhibit well-established efficacy in reducing sleep latency, improving total sleep time, and synchronizing circadian rhythms. These effects have been demonstrated in randomized controlled trials [118,223,224]. Agomelatine additionally shows antidepressant properties through 5-HT_2C_ receptor antagonism [136]. These drugs generally exhibit favorable safety profiles and minimal risk of dependence, which distinguishes them from conventional hypnotics such as benzodiazepines.

Conversely, herbal preparations such as *Matricaria chamomilla* (chamomile), *Melissa officinalis* (lemon balm), and *Nigella sativa* (black seed) have long been used in traditional medicine for their calming, anxiolytic, and sleep-enhancing properties. Their therapeutic effects are attributed to the presence of flavonoids, terpenoids, and other active compounds that modulate GABAergic and serotonergic neurotransmission, and possess antioxidant and anti-inflammatory properties [225,226]. Recent clinical research has provided encouraging evidence supporting the role of *Melissa officinalis* (lemon balm) in improving sleep quality. In a prospective, double-blind, placebo-controlled, cross-over study including 30 adults with self-reported sleep disturbances, a standardized *Melissa officinalis* phytosome extract significantly improved sleep quality, as assessed by the Pittsburgh Sleep Quality Index, compared to placebo. Participants reported shorter sleep latency, longer sleep duration, and better overall restfulness. Importantly, improvements also extended to secondary outcomes, including reduced daytime fatigue and enhanced mood [162]. Despite these benefits, the clinical evidence supporting the effects of herbal medicines remains limited, often derived from small-scale or pilot studies, and lacking robust placebo-controlled trials. Moreover, there is currently a lack of high-quality comparative studies directly evaluating herbal therapies against melatonergic drugs.

In a comprehensive systematic review and meta-analysis, Leach et al. [22] evaluated 14 randomized controlled trials involving 1602 participants and found that herbal medicines such as valerian, chamomile, kava, and wuling were no more effective than placebo or active comparators in improving sleep outcomes. While some studies reported modest benefits, the overall evidence remained inconclusive and statistically non-significant [22].

From a cost perspective, substantial differences favor herbal preparations and melatonin. In most European Union countries and the United States, melatonin in over-the-counter formulations represents an affordable therapeutic option, particularly at doses of 1–5 mg. In contrast, tasimelteon and ramelteon, as prescription-only medications, are associated with significantly higher treatment costs, which may restrict their widespread use, especially for long-term therapy. Herbal preparations available OTC are widely used in mild forms of insomnia and are preferred by patients seeking a natural approach.

Despite these advantages, key barriers to the broader application of phytotherapy remain, including the lack of standardization of active constituents, variability in commercial products, and the risk of herb–drug interactions. Further well-designed clinical trials are required to more clearly define the role of herbal therapies within therapeutic algorithms for insomnia. A summary of the main differences between melatonergic drugs and herbal preparations is presented in Table 5.

In summary, melatonergic agents and herbal preparations represent two distinct yet complementary approaches to insomnia management. While pharmacological agents offer well-established efficacy and regulatory validation, herbal therapies provide a promising yet underexplored alternative, particularly in mild cases or among patients preferring natural remedies. Given the growing interest in integrative medicine, future research should prioritize well-designed comparative trials, standardization of botanical extracts, and formal cost-effectiveness analyses to guide evidence-based clinical decision-making.

## 6. Conclusions

Melatonergic receptor agonists represent a safer alternative to classical hypnotics, particularly in the treatment of insomnia and circadian rhythm sleep disorders. However, their efficacy and scope of application remain heterogeneous. Melatonin, although safe and well-tolerated, displays low bioavailability and a short half-life, which limits its effectiveness in chronic insomnia. Ramelteon, a selective MT1/MT2 receptor agonist, has a favorable safety profile and poses no risk of dependence, yet its efficacy in extending total sleep time is modest. Tasimelteon has demonstrated efficacy primarily in Non-24-Hour Sleep–Wake Disorder, but its use outside this indication remains limited. Agomelatine, with its dual hypnotic and antidepressant action, offers clinical value, though its use is associated with a risk of hepatotoxicity. All reviewed agents undergo hepatic metabolism, primarily via cytochrome P450 enzymes, which introduces the potential for drug–drug interactions, especially in polypharmacy patients. Compared to benzodiazepines, melatonergic agonists exhibit a significantly better safety profile, lacking addictive potential, tolerance development, and cognitive impairment. Due to limited efficacy and pharmacokinetic variability, further research is warranted to develop novel MT1/MT2 agonists and to evaluate their utility in geriatric and psychiatric populations. Standardized plant-based preparations (e.g., valerian, passionflower), often used by patients as first-line therapies, should also be more extensively studied. Their clinical effects remain poorly documented, and the absence of standardization and potential interactions underline the need for further investigation into their efficacy and safety.

The study of the effects of medicinal plants on the mind and body is a long-standing tradition in medicine and pharmacology. However, using these plants to treat mood disorders, insomnia, stress, anxiety, depression, and related conditions is an under-explored area.

Due to their multiple constituents, plant-based medicinal products are a feasible alternative to drugs that target a single mediator (e.g., an enzyme). This mitigates non-specific toxicity and inhibits the development of drug resistance, which is most notable for minor, self-limiting conditions. Subsequent investigations must prioritize rigorous clinical trials of well-defined chemical preparations to assess the safety and efficacy of these herbal therapies. This will expedite their integration into conventional medical practice, elevating them from alternative treatments to mainstream therapies. Understanding the underlying mechanisms of these botanical interventions is essential for advancing pharmaceutical interventions, particularly for mental health issues. This will expand therapeutic options for various disorders and offer a more comprehensive, personalized approach to patient care. In conclusion, herbal and natural supplements may play a role in managing insomnia and sleep disorders; however, further research is needed to establish their efficacy, safety, and optimal use.

## Figures and Tables

**Table 1:** Efficacy of Melatonin in Sleep-Related Clinical Trials.

Population	TST/sTST (min)	SL/sSL (min)	Dose (mg)	References
40 patients ≥ 55 years with primary insomnia (20 PRM, 20 placebo)	PRM: 391.7 Placebo: 389.5	PRM: 13.7 Placebo: 22.6 (−9 min)	2 mg prolonged-release melatonin	[95]
Adults with primary insomnia, age 65–80 (PRM *n* = 137, Placebo *n* = 144)	After 3 weeks: +7.0 min After 6 months: +7.5 min	After 3 weeks: −15.6 min After 6 months: −14.5 min	2 mg prolonged-release melatonin	[96]
110 children (3–15 yrs) with chronić sleep problems	+22.4 min vs. placebo (sleep diaries, *p* = 0.04); +13.3 min vs. placebo (actigraphy, NS)	−37.5 min vs. placebo (sleep diaries, *p* < 0.0001); −45.3 min vs. placebo (actigraphy, *p* = 0.0003)	0.5–12 mg	[97]
40 children (6–12 yrs) with chronic sleep onset insomnia	+41 min	−63 min (diary), −75 min (actigraphy)	5 mg	[98]

**Table 2:** Efficacy of Ramelteon in Clinical Trials.

Population	TST (min)	SL/sSL (min)	oTST/sTST (min)	Dose (mg)	References
Adults ≥ 50 years with insomnia	+21 (objective)	−13.8	Not reported	4–8 mg	[131]
Adults with insomnia	+7.26 (objective)	−9.36/−4.3	+3.23 min (sTST), not statistically significant	4–32 mg	[134]
Adults with insomnia without comorbidities	+17.9 (oTST), +11.7 (sTST)	−14 (oSOL)/−8.74 (sSOL)	+2.02/+14.5 (long-term treatment)	4–16 mg (commonly 8)	[135]

**Table 3:** Efficacy of Agomelatine in Clinical Sleep Studies.

Population	TST/sTST (min)	SL/sSL (min)	Dose (mg)	Refrerences
Adults with MDD (*n* = 332)	↑ subjective sleep quality (LSEQ)	↑ getting to sleep (*p* = 0.001)	25–50	[139]
Adults with MDD (*n* = 138)	Preserved sleep cycles (vs. ↓ in escitalopram)	↓ sleep latency (*p* < 0.05)	25–50	[140]
Adults with depression (*n* = 7961)	Not reported	No increase in hypnotic use (*p* = 0.520)	25–50	[141]
Patients with alcohol dependence and insomnia (*n* = 9)	↓ PSQI score from 13.1 to 7.8	Improved sleep onset latency	25–50	[142]

(↑—increase, ↓—decrease).

**Table 4:** The list of medicinal herbs used in the treatment of insomnia.

Plant Name (Family)	Key Metabolites	Therapeutic Uses & Effects	Research Findings & Clinical Trials	Reference
*Matricaria chamomilla* L. (Chamomile); Asteraceae	Terpenoids, Phenolic compounds, Essential oils	Anxiolytic, anti-depressant, sleep quality improvement, potential in GAD and depression.	Clinical trials have shown effectiveness in treating anxiety and improving sleep quality.	[148,149,150,151,152,153,154,155]
*Melissa officinalis* L. (Lemon balm); Lamiaceae	Rosmarinic acid, Volatile metabolites: geranial, neral, citronellal, geraniol	Cognitive improvement, mood enhancement, sleep quality improvement.	Clinical trials have shown significant improvement in sleep quality and mood enhancement.	[16,156,157,158,159,160,161,162,163,164]
*Nigella sativa* L. (Black cumin); Ranunculaceae	Thymoquinone	Anti-anxiety properties, memory enhancement, stress management.	Animal trials show an increased sleep quality and reduced anxiety; human trials proved stress and sleep management	[165,166,167,168,169,170]
*Valeriana officinalis* (Valerian); Caprifoliaceae	essential oils, ketones, phenols, iridoid esters, valeric acid, aminobutyric acid, arginine, tyrosine, glutamine, as well as noncyclic, monocyclic, and bicyclic hydrocarbons	It has sedative and hypnotic properties, relieving anxiety and sleep disorders. It inhibits the uptake and stimulates the release of GABA, a partial agonist of the 5-hydroxytryptamine 2A receptor, which increases melatonin release.	Clinical trials have shown that Valerian was found to significantly improve sleep quality, anxiety, and depression symptoms in patients.	[171,172,173,174,175,176,177,178,179,180]
*Passiflora incarnata* (passionflower), Passifloraceae	alkaloids, phenolic compounds, flavonoids, and cyanogenic glycosides. The primary phytochemicals found in the passionflower are flavonoids (apigenin, luteolin, quercetin, and kaempferol) and flavonoid glycosides (vitexin, isovitexin, orientin, and isoorientin)	A sedative, anxiolytic, and hypnotic agent used to treat sleep disorders. It modulates the neurotransmitter system, primarily gamma-aminobutyric acid (GABA), serotonin, and the adrenergic system.	Clinical trials have shown that *Passiflora incarnata* (passionflower) regulates mood, anxiety and stress response	[181,182,183,184,185,186,187,188,189,190,191,192]
*Lavandula angustifolia* (Lavender)	Lavender oil contains linalyl acetate, linalool, α-aminobutyric acid	Used in aromatherapy, essential oils treat sleep disorders. Lavender’s scent is an anti-anxiety, anticonvulsant, analgesic, sedative, and sleep-inducing agent.	Clinical trials have shown that *L. angustifolia* relieves stress, reduces anxiety, and improves sleep quality in patients	[193,194,195,196,197,198,199,200,201,202,203,204,205,206,207,208,209,210,211]
Melatonin-containing plants	Melatonin	Activity of the plants resembles that of melatonin, and additionally, a marked antiradical and anti-inflammatory action supported by the complex of metabolites.	Melatonin was identified in several plant species, including *Coffea*, *Piper*, *Lycium*, *Brassica*, *Medicago*, *Chlorella* and *Oryza* species	[81,169,212,213,214,215,216,217,218,219,220,221,222]

**Table 5:** Comparison of melatonergic drugs and herbal preparations in the treatment of insomnia.

Criterion	Melatonergic Drugs	Herbal Preparations
Examples	Melatonin, Ramelteon, Tasimelteon, Agomelatine	*M. chamomilla*, *M. officinalis*, *N. sativa*
Mechanism of Action	MT1/MT2 agonism, 5-HT_2C_ antagonism (agomelatine)	GABA modulation, serotonin activity, antioxidant effects
Clinical Efficacy	High (multiple RCTs and meta-analyses)	Limited (mostly pilot trials)
Safety	High, low abuse potential	High, but risk of interactions and allergies
Use in Severe Insomnia	Confirmed by multiple trials	Not confirmed
Cost	Moderate to high	Low
Availability	Prescription only (except melatonin OTC in some regions)	OTC, dietary supplements
Additional Benefits	Antidepressant effects (agomelatine)	Anxiolytic, adaptogenic potential

## Abbreviations

The following abbreviations are used in this manuscript:

ICSD-3-TR	The International Classification of Sleep Disorders, Third Edition
ICD-11	11th Revision of the International Classification of Diseases
DSM-5	5th edition of the Diagnostic and Statistical Manual of Mental Disorders
SCN	Suprachiasmatic nucleus
LTP	Long-term potentiation
cAMP	Cyclic adenosine monophosphate
AC	Adenylate cyclase
PKA	Protein kinase A
P-CREB	Phosphorylated CREB
PKC	Protein kinase C
GC	Guanylyl cyclase
PLC	Phospholipase C
cGMP	Cyclic GMP
DAG	Diacylglycerol
IP_3_	Inositol trisphosphate
PVN	Paraventricular nucleus
VTA	Ventral tegmental area
OX1R/OX2R	Orexin-1-receptor/Orexin-2-receptor
GIRK	G protein–regulated inward rectifier
NMDA	N-methyl-D-aspartate
DMH	Hypothalamus
NREM	Non-rapid eye movement
REM	Rapid eye movement
VLPO	Ventrolateral preoptic nucleus
TMN	Tuberomammillary nucleus
LC	Locus coeruleus
LH/PF	Lateral hypothalamus/Perifornical area
GPCR	G protein-coupled receptor
NQO2	Quinone reductase 2
TST	Total sleep time
sTST	Subjective total sleep time
SL	Sleep latency
sSL	Subjective sleep latency
ASD	Autism spectrum disorder
LPS	Latency to persistent sleep
SE	Sleep efficiency
oSOL	Objective sleep onset latency
EMA	European Medicines Agency
MDD	Major depressive disorder
LSEQ	Leeds Sleep Evaluation Questionnaire
CGI	Clinical Global Impression
OR	Odds ratio
PSQI	Pittsburgh Sleep Quality Index
TCIM	Integrative medicines
WHO	World Health Organization
GAD	Generalized anxiety disorder
HRSD	Hamilton Rating Scale for Depression
GABA	Gamma-aminobutyric acid
NO	Nitric oxide
